# Acid-base adjustments and first evidence of denticle corrosion caused by ocean acidification conditions in a demersal shark species

**DOI:** 10.1038/s41598-019-54795-7

**Published:** 2019-12-19

**Authors:** Jacqueline Dziergwa, Sarika Singh, Christopher R. Bridges, Sven E. Kerwath, Joachim Enax, Lutz Auerswald

**Affiliations:** 1 Heinrich-Heine University, Düsseldorf, Institute of Metabolic Physiology/Ecophysiology, Düsseldorf, Germany; 2 Ocean and Coastal Research, Department of Environmental Affairs (DEA), Cape Town, South Africa; 3 Branch: Fisheries Management, Department of Agriculture, Forestry and Fisheries (DAFF), Cape Town, South Africa; 4 Department of Animal Sciences, Stellenbosch University, Stellenbosch, South Africa; 5 Institute of Inorganic Chemistry and Center for Nanointegration Duisburg-Essen (CeNIDE), University of Duisburg-Essen, Essen, Germany

**Keywords:** Animal physiology, Environmental impact

## Abstract

Global ocean acidification is expected to chronically lower the pH to 7.3 (>2200 µatm seawater *p*CO_2_) by the year 2300. Acute hypercapnia already occurs along the South African west and south coasts due to upwelling- and low-oxygen events, with increasing frequency. In the present project we investigated the impact of hypercapnia on the endemic demersal shark species *Haploblepharus edwardsii*. Specifically, we experimentally analysed acid-base regulation during acute and chronic hypercapnia, the effects of chronic hypercapnia on growth rates and on denticle structure- and composition. While *H. edwardsii* are physiologically well adapted to acute and chronic hypercapnia, we observed, for the first time, denticle corrosion as a result of chronic exposure. We conclude that denticle corrosion could increase denticle turnover and compromise hydrodynamics and skin protection.

## Introduction

The continuous absorption of anthropogenic CO_2_ from the atmosphere feeds the ongoing process of ocean acidification, affecting both the average CO_2_ levels and the magnitude of CO_2_ fluctuations^1,2^. The ongoing changes in the ocean’s carbonate chemistry caused by anthropogenic CO_2_ emissions are thought to have profound effects on the biology, distribution, morphology, behaviour and physiology of marine organisms^3–5^. The increased dissolution of CO_2_ in seawater results in a net increase in the concentration of H_3_O^+^ ions and a decrease in CO_3_^2−^ ions leading to a lowered pH - a process known as ocean acidification^6^. By the year 2300, a global pH of about 7.3 is expected^7^ as a result of an increased oceanic *p*CO_2_ of above 2200 µatm, with potentially severe effects on marine organisms and ecosystems. Elucidating these effects is paramount to predict and mitigate against their impact.

The South African west and south coasts are influenced by the environmental dynamics in the Benguela Large Marine Ecosystem (BCLME), characterised by coastal upwelling and, in some areas, periods of low-oxygen due to algal decay and bacterial respiration. The BCLME is one of the world’s largest Eastern Boundary (upwelling) systems^8^. Frequent upwelling events in austral summer cause periodic episodes of hypercapnia (pH levels 7.4–7.6) which can even reach pH 6.6 for several days during low oxygen events in autumn^10^. Upwelling takes place in 3–10 day cycles in spring and summer^9^, moving cold (~10 °C), hypercapnic water (pH 7.4–7.6) closer to the surface^11,12^. Low oxygen events normally occur after the upwelling season caused by the collapse of phytoplankton blooms^13^. As a result of climate change, upwelling events in eastern boundary current systems, and in turn hypercapnic episodes, are predicted to become longer, more frequent and severe in the near future^14–17^, with much variability observed in the BCLME^18^.

Cartilaginous fishes play an essential role in the global marine ecosystems^19^ and some species represent an important fishing resource^20^, yet the consequences of ocean acidification for this phyletic group remain poorly understood^21^. Their slow rate of evolution, low phenotypic plasticity and low adaption potential due to long generation times and low fecundity put sharks at a higher risk from the effects of climate change^22^. The South African chondrichthyofauna includes representatives from all 10 orders of cartilaginous fishes, 44 of 60 families (73%), 100 out of 189 genera (53%), and over 181 of the 1171 world species (15%), with a high degree of endemism^23^. The puffadder shyshark (*Haploblepharus edwardsii*, Scyliorhinidae) is a small (600 mm max. total length), benthic catshark species endemic to South African shallow temperate waters. Puffadder shy sharks are generalist feeders residing in kelp beds and sand-inundated reefs, preying on crustaceans, polychaetes and small teleosts in relatively shallow water. In its habitat, *H. edwardsii* is exposed to highly variable pH and temperature conditions.

There have been a number of studies investigating the effects of hypercapnia on invertebrates^24^ and teleosts^25,26^, but very few on chondrichthyans^27^. Physiological effects such as haemolymph- or blood acidosis are known for invertebrates and fishes^3,26,28^. Effects on growth rates have been found to be highly variable among different groups and life history stages^29^. Recent findings suggest that embryo survival and development time are unlikely to be affected, but effects on body condition, growth, aerobic potential and behaviour have been suggested for demersal shark species^27^. Effects on formation and alteration of skeletal, otolith or integument structures have been elucidated in various studies^30^. The potential impact of hypercapnia on mineralisation in elasmobranchs has been less studied so far: Chronic hypercapnia has an impact on the skeleton of juvenile (i.e. developing) skates^31^ and a minor effect on mineralisation of vertebrae of juvenile sharks^32^. When the impact of hypercapnia on chondrichthyan denticles was investigated, no effects were found in juvenile catshark^21^ (4–6 months at 1100 µatm) and skate embryos^31^ (1 month at 990 µatm). Generalisation of existing results is difficult due to the diversity of experimental conditions and species’ responses, but species in highly variable environments might already reside close to their biological limits^24^ and rapid change of baseline conditions, such as predicted for the BCLME, might result in suboptimal and even lethal conditions.

Here we investigate effects of future ocean acidification levels on *H. edwardsii*. The hypercapnic level (pH 7.3, expected by year 2300 according to IPCC scenario) was chosen to be below the range of what occurs regularly during upwelling and taking into account that pH levels are usually well below the global average pH of 8.1 in the BCLME. Puffadder shysharks are already adapted to a highly variable environment and are restricted in their distribution to the Southern tip of Africa without a possibility of a range shift to mitigate against negative effects of climate change. The species is well suited for our experiments because they are relatively small, easy to rear and can easily be obtained in relevant numbers for experimentation. In the present study we specifically investigated the acid-base regulation during the exposure of puffadder shysharks to (1) acute (32 h) and (2) chronic (9 weeks) hypercapnia in laboratory experiments. Furthermore we examined the effects of chronic hypercapnia on (3) growth rates and (4) on denticle structure by means of Scanning Electron Microscope (SEM) scans and elemental composition analysis. We hypothesised that puffadder shy sharks, due to their environmental adaptation, are able to physiologically acclimatize to acute and possibly chronic hypercapnia. We further hypothesize that physiological compensation during chronic hypercapnia will come at an energetic cost that decreases somatic growth. Finally, we postulate that the low pH will have a detrimental effect on the denticle structure of the shy shark, similar to human dental corrosion after exposure to carbonated drinks.

## Materials and Methods

### Experimental animals

All research presented here was conducted under permission of the Research Ethics Committee: Animal Care and Use of Stellenbosch University (SU-ACUM14-00006) and is in accordance with the relevant guidelines and regulations. Specimens (80) of the Puffadder shyshark *H. edwardsii* were caught in the harbour basin of the False Bay Yacht Club in Simons Town, South Africa (34.07°S, 18.33°E) in austral spring. Sharks were caught by SCUBA divers by hand after setting out bait (sardines) in perforated 5 l plastic bottles. Caught sharks were collected in nets until they were transferred into an 800 l tank on a car trailer. Water in the tank was continuously provided with oxygen (technical) from a cylinder. After capture, sharks were transported within an hour to holding tanks at the Research Aquarium of the Department of Agriculture, Forestry and Fisheries (DAFF) in Cape Town.

In Cape Town, they were weighed and maintained in round flow-through holding tanks (4500 l) for four months prior to experimentation (pH ranged from 7.9 to 8.1; T_A_ from 8.4 to 16.8 °C). They were fed rations of 5% of average body mass with pieces of squid once a week. Sharks were not fed in the week of experimentation.

### Experimental procedures

For acute exposure, 66 larger sharks (179 ± 52 g, 67% male) were acclimatized for 48 h prior to experimentation in smaller round tanks (∅ = 1.2 m, h = 1 m, 1130 l). Tanks were well mixed by propellers and aerated by compressed air. At the start of the trial, individuals were distributed between two replicate control- (normocapnic) or hypercapnic tanks (141 l rectangular glass tanks) with corresponding pH levels of ~7.3 and ~8.0, respectively. Weight of animals did not differ significantly between replicate groups (ANOVA, p = 0.993). For blood sampling, individuals were removed from tanks at the respective time points of incubation, i.e. after 1.5, 3, 6 and 24 hours, alternating between replicate tanks. Each time point represents samples from both replicates of the same treatment (3 per replicate for hypercapnic treatment, 2 or 3 for normocapnic treatment. For the starting value (time 0), blood was collected from sharks from the same batch. These sharks were not incubated thereafter. To test recovery, some sharks were transferred after 24 h of exposure into tanks with normocapnic conditions and sampled after a further 8 h (i.e. a total of 32 h from the start of the trial). After sampling, sharks were transferred back into the acclimation tank and not used further. Blood was sampled (see below) only once from each shark. Analyses were carried out in the statistical software environment R (Ver. 3.0.1.), including the nlme package (Ver. 3.0.1.)^33^. Differences between parameters were tested for each interval against the base value (0 h) for the normocapnic (control) group and the hypercapnic (treatment) by means of ANOVA. Response variables were modelled as a function of the interaction between sampling time and treatment with linear-mixed effects models. *Tank* was initially included as a random effect, but found to increase the AIC value when tested against a fixed-effects model and consequently dropped. Filtered seawater for the system was provided by the main water storage tank of the research aquarium. It supplied each replicate via a header (mixing) tank. In two of the header tanks, pH was adjusted by its own CO_2_ supply for hypercapnic treatment. This was accomplished by using a pH controller (7074/2, TUNZE, Germany) containing a solenoid valve (7074.111) and a pH electrode (7070.110) attached to a 9 kg CO_2_ bottle (technical). A level of pH of 7.3 was selected as this level is predicted by the year 2300^7^. The experiment was carried out in a room with stable ambient air temperature (ranging from 16–18 °C) so that additional control of seawater temperature was unnecessary. During the acute experiment, seawater conditions were tested five times in each replicate tank (summarised in Table 1) and did not differ significantly between replicates of each treatment (pH, T; ANOVA).

**Table 1:** Physicochemical seawater parameters recorded during acclimation, normocapnia, hypercapnia and recovery in acute* and chronic* experimental treatments of adult *H. edwardsii*.

Treatment	T_A_ °C	pH_w_	A_T_ µmol kg^−1^	O_2_%	Salinity ‰	Ca^2+^ mmol l^−1^	Mg^2+^ mmol l^−1^	*p*CO_2_ Torr (µatm)	HCO_3_^−^ mmol l^−1^	CO_3_^2−^ mmol l^−1^
**Acute exposure**										
Acclimation	17.8 ± 0.0	8.05 ± 0.02	2000 ± 3	94.7 ± 0.1	34.9 ± 0.0	10.1 ± 0.4	52.3 ± 4.3	0.3 ± 0.0 (337 ± 15)	1.7 ± 0.0	0.2 ± 0.0
Normocapnia	17.3 ± 1.0	7.99 ± 0.07	1963 ± 22	91.8 ± 0.3	34.9 ± 0.0	11.2 ± 0.4	54.0 ± 2.1	0.3 ± 0.0 (386 ± 8)	1.7 ± 0.0	0.1 ± 0.0
Hypercapnia	17.0 ± 1.1	7.31 ± 0.06	2010 ± 50	90.0 ± 0.0	35.0 ± 0.0	10.7 ± 0.1	52.7 ± 1.4	1.7 ± 0.0 (2184 ± 45)	2.0 ± 0.0	0.0 ± 0.0
Recovery	17.2 ± 0.2	8.01 ± 0.01	1991 ± 12	92.3 ± 0.2	35.0 ± 0.0	10.9 ± 1.0	53.2 ± 2.3	0.3 ± 0.0 (371 ± 9)	1.7 ± 0.0	0.2 ± 0.0
**Chronic exposure**										
Normocapnia	16.4 ± 0.0	7.93 ± 0.06	1900 ± 300	90.7 ± 0.2	35.0 ± 0.0	10.8 ± 0.2	52.4 ± 1.9	0.3 ± 0.0 (437 ± 11)	1.7 ± 0.2	0.1 ± 0.0
Hypercapnia	16.7 ± 1.1	7.36 ± 0.05	1980 ± 60	90.1 ± 0.6	35.0 ± 0.0	11.0 ± 0.1	53.5 ± 2.0	1.4 ± 0.0 (1904 ± 22)	1.9 ± 0.0	0.0 ± 0.0

*Acute treatment: 32 h including 8 h recovery; chronic treatment: 9 weeks.

Subsequently, the remaining 13 smaller sharks were taken from the holding tank (see above) for chronic exposure. They were weighed (*w*) total length (*L*) measured and tagged (barbed dart tags (D-tag; 89 mm, Ø 1.4 mm; Hallprint Pty Ltd, South Australia) left of the first dorsal fin (121.4 ± 34.2 g, 85% male, see Results for more details). Thereafter they were transferred into normocapnic- or hypercapnic replicate tanks as described above, except that rectangular 1000 l plastic tanks were used here. All tanks were well mixed and aerated. ANOVA revealed no difference in *w* and *L* between the four replicate groups (p = 0.993; 0.914). In the two weeks prior to chronic exposure, seawater temperature in the holding tanks was in the range of 15.7–16.3 °C. Sharks of both treatments were acclimatized for a week to around 18 °C after which the pH for hypercapnic treatment was lowered in two steps in five days from approximately 8.1 to 7.3, using a pH control system as described above. The experimental pH of 7.3 was selected as this level is predicted to be reached by the year 2300^7^. It will possibly be reached earlier in the BCLME as it is close to values already attained over short periods during severe upwelling periods and after decay of algal blooms^10^. Seawater temperature was allowed to fluctuate with the incoming seawater. Similar seawater temperatures were recorded in all replicate tanks throughout experimentation. Seawater parameters were measured daily with the exception of A_T_ which was measured thrice a week (summarised in Table 1) and did not differ significantly between replicates of the same treatment (pH, T; ANOVA). Sharks remained under experimental conditions for 63 days (~9 weeks). To record growth and to adjust the food rations, sharks were re-weighed and measured after 4, 6 and 9 weeks. Paired t-tests were carried out to test for significant changes in length and mass within treatments. Differences at the end of incubation (blood parameters, elemental composition and physical damage of denticles) were modelled with linear-mixed effects models that included treatment as fixed and *Tank* as random effect. Equally to the acute experiment, *Tank* was found to increase the AIC value when tested against a fixed-effects model and consequently dropped (R Ver. 3.0.1.)^33^.

Seawater *p*CO_2_, [CO_3_^2−^] and [HCO_3_^−^] were calculated using measured pH, salinity, ambient temperature (T_A_) and total alkalinity (A_T_)^34^ as constants in CO2SYS software^35^. Oxygen concentration was determined using a Multi 350i meter set (WTW, Germany). Water quality was monitored by measuring NH_3_ concentration (Ammonia test kit, Sera, Germany) and never exceeded 0.09 mg l^−1^.

### Sampling

At the given intervals during the acute experiment and at termination of the chronic experiment, sharks were removed from their tanks, heads (eyes) were covered by a seawater-soaked cloth to reduce stress and prevent curling of the tail and animals placed upside down on a seawater-soaked cloth. In addition, head and tail were held tight by hand to avoid movement. Approximately 1 ml blood was immediately withdrawn from the caudal vein by syringe with a hypodermic needle (Neomedic 1 ml, 26 G) into a 2 ml syringe treated with heparin before the animals were carefully returned to the tank. All animals from the chronic experiment were subsequently sacrificed using ethylene glycol monophenyl ether (C_8_H_10_C_2_, 0.8 ml l^−1^). Skin samples were then taken dorso-laterally next to the first dorsal fin and frozen at −20 °C for electron-microscopic- and elemental analysis.

### Analysis of denticles

Micrographs of shark skin areas and denticles were obtained by scanning electron microscopy (SEM) using a Leo 1430VP (Zeiss, Germany) of gold-platinum-sputtered samples whereas elemental composition of the outer denticle surface was analysed using energy-dispersive X-ray spectroscopy (EDX) with an ESEM Quanta 400 FEG instrument (Thermo Scientific, USA) after sputtering with gold and palladium (80:20)^36,37^. On the resulting SEM micrographs, ratios of damaged and intact denticles were quantified by counting.

### Blood acid-base balance

The blood pH was measured within 20 s after sampling using an Orion 3 star pH meter equipped with an Orion 8220 BNWP micro pH electrode (Thermo Scientific, USA). Calibration was performed with NBS precision buffers (Applichem, Germany) at the same temperature as that of ambient seawater of the experimental tanks. A blood subsample (50 µl) was immediately injected into a de-gassing (magnetic stirrer) chamber containing 200 µl of 100 mM H_2_SO_4_ and liberated total CO_2_ (cCO_2_) determined as described previously^38^. From measured pH and cCO_2_ values, *p*CO_2_, and [HCO_3_^−^] were calculated using derivatives of the Henderson Hasselbalch equation (I and II). The required solubility coefficient αCO_2_ and dissociation constant pK’_1_ of carbonic acid were obtained from Boutilier *et al*.^39^ for catsharks (*Scyliorhinus canicula* and *S. stellaris*).

1 $$pC{O}_{2}=\frac{cC{O}_{2}}{{10}^{pH-p{K^{\prime} }^{1}}\times \alpha C{O}_{2}+\alpha C{O}_{2}}$$

2 $$HC{O}_{3}^{-}=cC{O}_{2}-\alpha C{O}_{2}\times pC{O}_{2}$$

Ca^2+^ and Mg^2+^ concentrations were determined spectrophotometrically by commercial kits (Diaglobal, Germany) in undiluted small subsamples.

### Haematocrit

Subsamples of 500 μl blood were immediately transferred into an EDTA pre-treated K2E reaction vessel (BD Microtainer, USA) for measurement of haematocrit. The vessels were closed and the samples shaken to ensure mixing of EDTA. Thereafter, 80 iu/ml sodium-heparinised micro haematocrit capillaries (Marienfeld, Germany) were completely filled, sealed with plasticine and subsequently spun at room temperature for 5 min in a Haematospin 1300 centrifuge (Lasec, SA). Haematocrit was subsequently quantified using a Micro Haematocrit reader (Hawksley, UK).

## Results

### Blood acid-base balance during acute hypercapnia

The acute exposure experiment revealed differences between responses to normocapnic and hypercapnic conditions, respectively.

**Figure 1:**
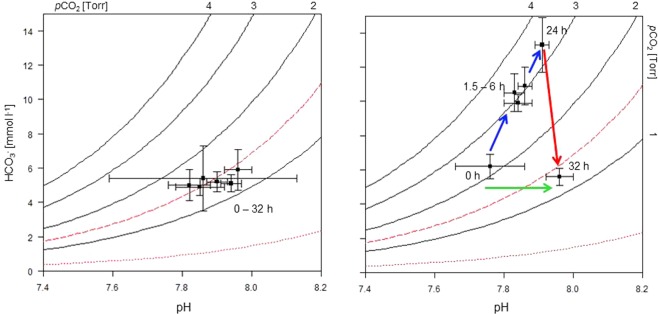
Henderson-Hasselbalch (pH-bicarbonate) diagrams for blood of *H. edwardsii* constructed from the time course of values during acute exposure presented in Table 2. Left panel: during 24 h normocapnia and subsequent 8 h recovery (n = 5), right panel: during 24 h hypercapnia followed by 8 h recovery (n = 6). Values are means ± S.D.; *p*CO_2_ isopleths were derived from the Henderson-Hasselbalch equation. Values for the first dissociation constant (pK’_1_) and Solubility coefficient (αCO_2_) were derived from Boutilier *et al*.^31^ and were: pK’_1_ = 6.01, αCO_2_ = 0.044 (18 °C). Dashed line = normocapnic seawater isopleth, dotted line = hypercapnic seawater isopleth (1 Torr = 0.133 kPa). Values are means ± S.D. Arrows indicate course of bicarbonate buffering from start to 24 h hypercapnic incubation (blue) and recovery following 24 h of incubation (red). Green arrow indicates alkalosis.

In the normocapnic control group, there were no significant changes in pH (Table 2). Extracellular total CO_2_ (cCO_2_) levels remained in a narrow range of between 5 and 6 mmol l^−1^ throughout the course of the experiment. Accordingly, calculated values of [HCO_3_^−^ + CO_3_^2−^] and *p*CO_2_ showed very little change. This is also evident from the Henderson-Hasselbalch diagram (Fig. 1).

**Table 2:** Time course of *in vivo* blood parameters of adult *H. edwardsii* during acute exposure to normocapnic and hypercapnic seawater conditions.

Exposure time	n	pH	cCO_2_	*p*CO_2_		[HCO_3_^−^ + CO_3_^2−^]	Ca^2+^ mmol·l^−1^	Mg^2+^ mmol·l^−1^
			mmol·l^−1^	Torr	(kPa)	mmol·l^−1^		
**Normocapnia (h)**								
0	5	7.90 ± 0.03	5.3 ± 0.6	1.5 ± 0.1	(0.2 ± 0.0)	5.2 ± 0.6	7.9 ± 0.9	3.5 ± 0.2
1.5	5	7.85 ± 0.07	5.0 ± 0.5	2.1 ± 0.5	(0.2 ± 0.0)	4.9 ± 0.5	7.7 ± 1.6	3.4 ± 0.1
3	5	7.82 ± 0.06	5.1 ± 0.9	1.7 ± 0.6	(0.2 ± 0.1)	5.0 ± 0.9	7.5 ± 0.7	3.3 ± 0.3
6	5	7.86 ± 0.27	5.5 ± 1.9	1.7 ± 0.5	(0.2 ± 0.1)	5.4 ± 1.9	7.8 ± 1.1	3.4 ± 0.3
24	5	7.96 ± 0.04	6.0 ± 1.2	1.5 ± 0.3	(0.2 ± 0.0)	5.9 ± 1.2	8.2 ± 1.0	3.6 ± 0.4
32 (Recovery)	5	7.94 ± 0.03	5.2 ± 0.5	1.3 ± 0.2	(0.2 ± 0.0)	5.1 ± 0.5	8.1 ± 0.8	3.4 ± 0.3
**Hypercapnia (h)**								
0	6	7.76 ± 0.10	6.3 ± 0.7	2.4 ± 0.4	(0.3 ± 0.1)	6.2 ± 0.7	7.5 ± 0.9	3.2 ± 0.3
1.5	6	7.83 ± 0.03*	10.6 ± 1.1*	3.4 ± 0.2*	(0.4 ± 0.0)	10.5 ± 1.1*	7.4 ± 1.5	3.2 ± 0.1
3	6	7.86 ± 0.02*	11.0 ± 1.1*	3.3 ± 0.3*	(0.4 ± 0.0)	10.9 ± 1.1*	6.6 ± 1.0	3.2 ± 0.3
6	6	7.84 ± 0.04*	10.0 ± 0.5*	3.1 ± 0.2*	(0.4 ± 0.0)	9.9 ± 0.5*	7.2 ± 1.2	3.3 ± 0.1
24	6	7.91 ± 0.02*	13.5 ± 1.6*	3.6 ± 0.4*	(0.5 ± 0.1)	13.3 ± 1.6*	8.9 ± 0.8	3.6 ± 0.2
32 (Recovery)	6	7.96 ± 0.04*	5.7 ± 0.5	1.4 ± 0.20	(0.2 ± 0.0)	5.6 ± 0.5	8.0 ± 1.6	3.3 ± 0.2

Values are means ± S.D. *Significantly (p < 0.05) different treatment: time interaction.

Despite the increase in seawater *p*CO_2_ and subsequently blood *p*CO_2_ (Table 2), the extracellular pH in sharks in the hypercapnic treatment increased by 0.1 units in the first 3 h after sudden exposure to hypercapnia (Table 2). From this point, it followed a similar course as observed in normocapnic sharks (see above). To the 24 h mark, this comprises an over-compensation of pH by approximately 0.15 pH units or reduction in [H^+^] by 5.1 mM (29%) compared with initial levels. pH increased by another 0.05 units during the subsequent 8 h of recovery in normocapnic seawater. All treatment: time interactions were significant from 1.5 h incubation onwards.

Extracellular cCO_2_ levels rose by 114% from an initial 6.3 mmol l^−1^ during 24 h of hypercapnic exposure (Table 2). These increases were significant from 1.5 h incubation onwards. Subsequent recovery in normocapnic seawater caused a sharp decline by 58% (not sig. different to 0 h) to below the initial level. Values for [HCO_3_^−^ + CO_3_^2−^] followed a similar trend (Table 2). The partial pressure of CO_2_ increased from its initial level peaking at the 24 h time interval (sig. different from 1.5 h incubation) after which it decreased sharply to below the initial value during recovery (not sig.). Data was used to construct a Henderson-Hasselbalch diagram, depicting the extracellular pH, calculated [HCO_3_^−^ + CO_3_^2−^] and *p*CO_2_ values (Fig. 1). No acidosis occurred initially after exposure to hypercapnia which would have been indicated by a shift to the left. Compensation by an increase in [HCO_3_^−^ + CO_3_^2−^] started immediately, buffering the blood and leading to an alkalosis when compared to the initial pH measured. This is indicated by a shift to the right. This alkalosis is carried over to the 32 h (recovery) value although *p*CO_2_ and [HCO_3_^−^ + CO_3_^2−^] dropped sharply during this period. In contrast, there is very little change in sharks exposed to normocapnic conditions (Fig. 1). Between treatments, haemolymph pHs only differed up to the 1.5 h time interval, after which both followed a similar path, including 8 h of recovery (Table 2). Total CO_2_, calculated *p*CO_2_ and [HCO_3_^−^ + CO_3_^2−^], however, were substantially higher in hypercapnic sharks with the exception of the recovery values. Concentrations of Ca^2+^ and Mg^2+^ were stable in both treatments and did not differ between treatments throughout the experiment (Table 2).

### Acid-base balance and other blood parameters during chronic hypercapnia

**Figure 2:**
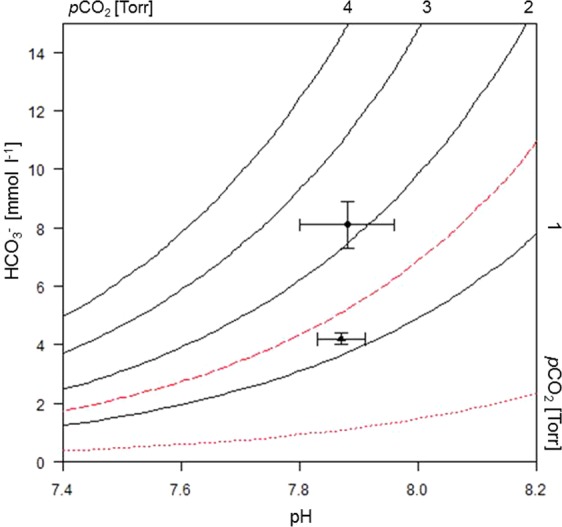
Henderson-Hasselbalch (pH-bicarbonate) diagram for blood of *H. edwardsii* after experimental exposure of 9 weeks. Values are for sharks kept in normocapnic conditions (lower data point; n = 7) and those exposed to hypercapnic conditions (upper data point; n = 6). *p*CO_2_ isopleths were derived from the Henderson-Hasselbalch equation. For calculation details see Fig. 1 and Materials & Methods.

The pH levels measured in the blood of hypercapnic- and normocapnic incubated sharks were identical (not sig.), whereas cCO_2_ had approximately doubled (sig.) to 8.3 mM under hypercapnia compared with normocapnia (Table 3). Accordingly, calculated *p*CO_2_ and [HCO_3_^−^ + CO_3_^2−^] were 2.2 Torr compared to 1.1 Torr (not sig.) and bicarbonate 8.1 mM compared to 4.2 mM under normocapnia (sig.), respectively. Water *p*CO_2_ levels rose from 0.3 to 1.4 Torr during hypercapnic incubation but venous blood was significantly higher at 2.2 Torr. The Henderson-Hasselbalch diagram provided an illustration of the interaction of extracellular pH, calculated haemolymph bicarbonate and *p*CO_2_: At similar pHs in sharks from both treatments, elevation of *p*CO_2_ and [HCO_3_^−^ + CO_3_^2−^] caused a vertical shift in hypercapnic sharks, indicating bicarbonate buffering that results in slight alkalosis (Fig. 2). [Ca^2+^] and [Mg^2+^] were at the same level after both treatments (not sig.; Table 3). Haematocrit levels were similar after chronic treatment and not sig. different (Table 3).

**Table 3:** *In vivo* blood parameters of adult *H. edwardsii* after exposure to normocapnic and hypercapnic seawater conditions for nine weeks.

Exposure time	n	pH	cCO_2_ mmol·l^−1^	*p*CO_2_		[HCO_3_^-^ + CO_3_^2−^] mmol·l^−1^	Ca^2+^ mmol·l^−1^	Mg^2+^ mmol·l^-1^	Haematocrit %
				Torr	(kPa)				
Normocapnia (h)	7	7.87 ± 0.04	4.3* ± 0.2	1.1 ± 0.2	(0.1 ± 0.0)	4.2* ± 0.2	8.1 ± 0.8	3.6 ± 0.1	30 ± 5
Hypercapnia (h)	6	7.88 ± 0.08	8.3 ± 0.9	2.2 ± 0.7	(0.3 ± 0.1)	8.1 ± 0.9	8.8 ± 0.8	3.6 ± 0.2	27 ± 3

Values are means ± S.D. (n = 6–7). *Significantly different between treatments (p < 0.05).

### Analysis of denticles following chronic hypercapnia

**Figure 3:**
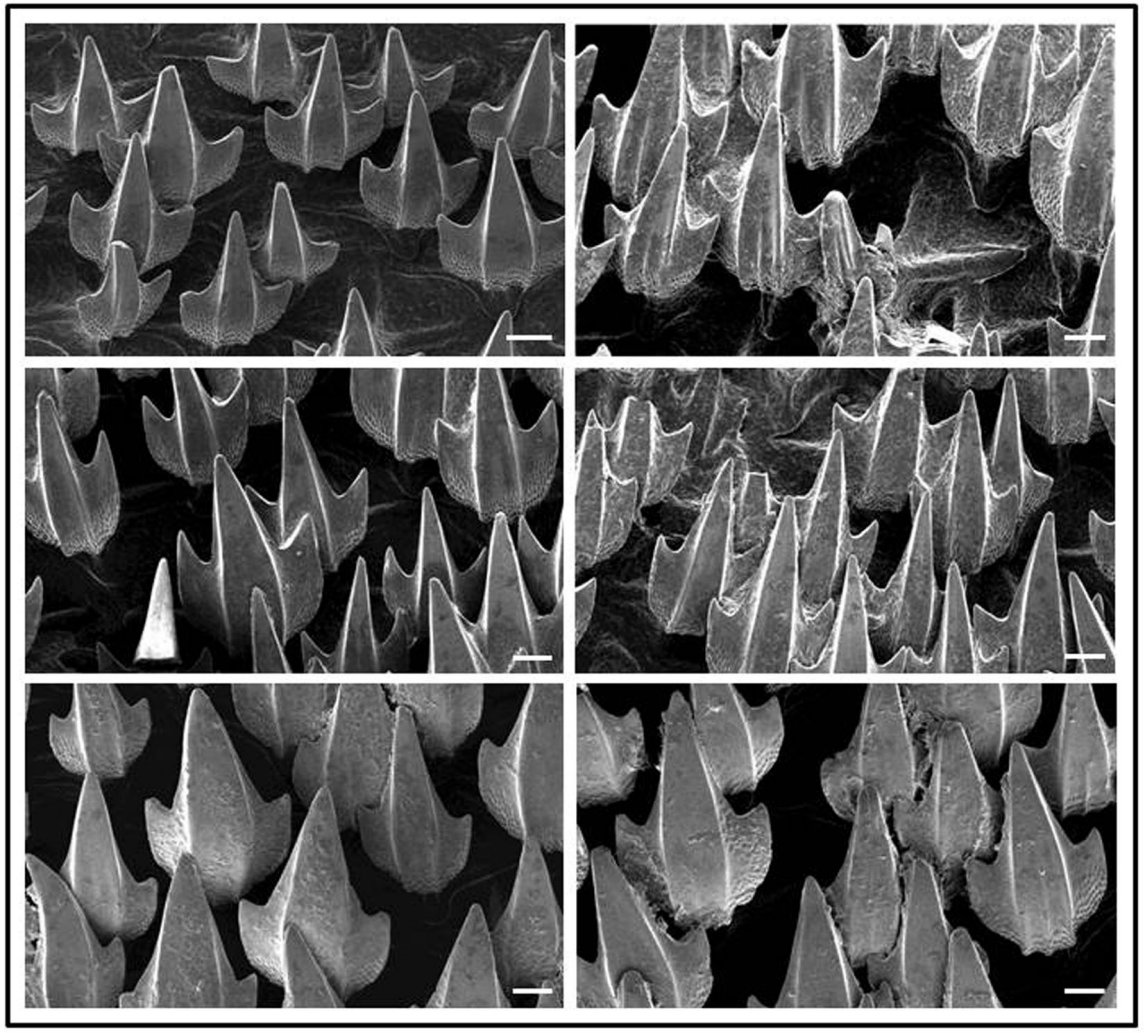
SEM observations of a defined skin area, populated by denticles, from individual *H. edwardsii* after experimental exposure of 9 weeks. Groups of denticles from 3 sharks that were kept in normocapnia are depicted in panels of the left column, those from hypercapnia in the right column. Size bars indicate 100 μm.

**Figure 4:**
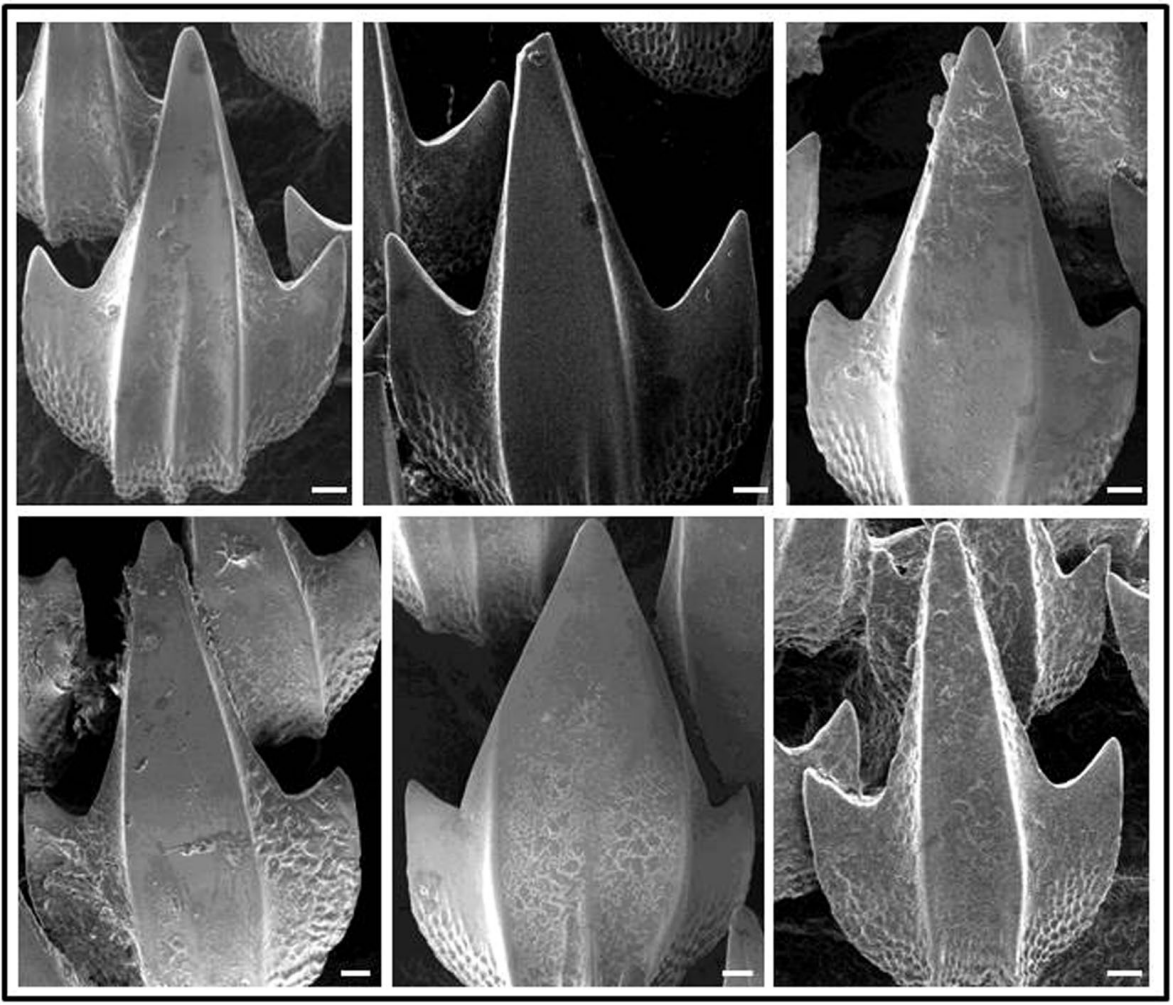
Close-up SEM view of select denticles from individual *H. edwardsii* after experimental exposure of 9 weeks. Single denticles from 3 sharks (same as in Fig. 3) that were kept in normocapnia are depicted in panels in the top row, those from hypercapnia in the bottom row. Size bars indicate 30 μm.

SEM analysis of skin areas covered with denticles revealed contrasting results: Denticles from normocapnic sharks were mostly intact and had a shiny surface with sharp edges (Fig. 3). In contrast, many denticles from hypercapnic sharks were damaged and their surface appears corroded and edges less sharp (Fig. 3). Comparison of SEM images from individual denticles confirmed this. Surfaces look smoother on normocapnic denticles (Fig. 4). Quantitative analysis of denticles from SEM micrographs revealed that significantly less (n = 3, p < 0.05) denticles (9.2 ± 3.2%) were damaged (pieces broken off) on normocapnic sharks than on hypercapnic ones (25.0 ± 6.7%).

Elemental composition of denticles from both treatments revealed some significant differences: Two of the elements that form fluoroapatite (Ca_5_(PO_4_)_3_F) and hydroxyapatite (Ca_5_(PO_4_)_3_OH) that are normally present in substantial quantities, Ca (−35%) and P (−26%), have a lower proportion in denticles from hypercapnic sharks (sig.), resulting in a lower Ca: P ratio (Table 4). In contrast, the proportions of C (+29%) and O (+15%,) are elevated although not significantly in the case of O.

**Table 4:** Elemental composition (in wt %) of denticles from adult *H. edwardsii* after exposure to normocapnic and hypercapnic conditions for nine weeks.

Element	C	N	O	F	Na	Mg	P	Au	Cl	Pd	Ca	Ca: P
**Normocapnia** (n = 5)	24.84 ± 4.40	3.34 ± 0.66	26.98 ± 4.48	0.59 ± 0.11	0.79 ± 0.10	0.23 ± 0.09	11.60 ± 2.08	5.57 ± 1.18	0.43 ± 0.15	1.62 ± 0.40	24.04 ± 6.15	2.05 ± 0.21
**Hypercapnia** (n = 4)	31.99 ± 2.66	4.05 ± 0.15	30.99 ± 3.87	0.52 ± 0.24	0.79 ± 0.03	0.32 ± 0.18	8.60 ± 0.83	5.26 ± 0.89	0.35 ± 0.35	1.59 ± 0.36	15.56 ± 1.95	1.80 ± 0.08
% difference to normocapnia	28.8*	21.3	14.9	−12.0	0.6	38.0	−26.2*	−5.6	−17.6	−1.4	−35.3*	

Values are means ± S.D. *Significantly different from normocapnia group (Students t-test; p < 0.05).

### Impact of chronic hypercapnia on body size

Sharks from the chronic normocapnic treatment increased in total length by 0.2 ± 0.6 cm (0.5%) from 31.6 ± 2.8 to 32.4 ± 3.0 cm and by mass by 7.7 ± 9.9 g (6.2%) from 123.8 ± 33.4 to 131.5 ± 37.1 g. Total length of hypercapnic sharks remained relatively stable (+0.1 ± 0.5 cm = 0.2%) from 31.4 ± 2.1 to 31.5 ± 2.1 cm and their mass changed by 0.8 ± 4.5 g (0.7%) from 118.5 ± 27.6 to 119.3 ± 29.0 g in nine weeks. The changes in length and mass were neither significantly different within nor between treatments.

## Discussion

The main findings of the present study are that (1) *Haploblepharus edwardsii* adjusts well physiologically (i.e. regulation of acid-base balance) to acute hypercapnia. (2) This regulation can be maintained during chronic hypercapnic exposure. (3) The prolonged regulation is likely to be energetically costly but in the present study no significant depression of somatic growth was observed. (4) Although the sharks can maintain their acid-base balance, prolonged exposure to hypercapnia has detrimental chemical effects that cannot be compensated, namely the dissolution of their denticles’ surface.

As a result of upwelling, the habitat of *H. edwardsii* is characterised by short periods of strong hypercapnia. Acute environmental hypercapnia results in increased extracellular *p*CO_2_ and decline of pH and, if uncompensated, would most likely impact gas exchange of haemoglobin and at the tissues. Compensation mechanisms exist in many marine animals in the form of an elevation of bicarbonate [HCO_3_^−^] levels to return pH levels to close to original values^3^. Also, the outward gradient for CO_2_ removal would become difficult to maintain due to an insufficiently high extracellular *p*CO_2_ level compared with the increased environmental *p*CO_2_. Such compensatory capacity is present in *H. edwardsii*.

We could demonstrate that *H. edwardsii* possesses the necessary compensation mechanisms to react to a sudden onset of hypercapnia: A rapid elevation of [HCO_3_^−^] by a net 7.1 mmol l^−1^ (+115%) after 24 h was observed, with a near doubling (+4.3 mmol l^−1^) already after 90 min of exposure, likely rapid enough to prevent a decline of arterial plasma pH. At the same time, the arterial plasma *p*CO_2_ was elevated by a net 1.2 Torr (+50%). This increased the outward CO_2_ gradient to 1.9 Torr, despite elevation of ambient *p*CO_2_ by 1.4 Torr. The 1.7 Torr of the latter was well within the range of resting plasma *p*CO_2_ and would have made gas exchange impossible. In the normocapnic group, the gradient was 1.2 Torr after the same incubation period. Such fast response is indicative of exposure and adaptation of the species to frequently elevated hypercapnia in its habitat^40^.

The Henderson-Hasselbalch diagram (Fig. 1) illustrates the interaction of arterial plasma pH, calculated plasma bicarbonate and *p*CO_2_: Whereas values are concentrated in a very restricted area throughout entire experimentation in the normocapnic group, the situation is very different in the hypercapnic group. Over-compensation of plasma pH (move to right, alkalosis) due to the elevation of bicarbonate levels (upward move) caused an increase in pH. In other species, a transitional acidosis (drop in plasma pH) that lasted several hours, has been observed before compensation through bicarbonate became effective^41,42^. In our study, a further increase (along a constant *p*CO_2_ isopleth) was caused by bicarbonate increase until 24 h of exposure. Such rapid responses indicate the presence of an efficient Cl^−^/HCO^3−^ exchanger as was previously found in the gills of sharks^43^, possibly supplemented by a Na^+^/NH_4_^+^ exchanger^42^. In addition, an involvement of a Na^+^-K^+^-ATPase as in teleosts is possible^44^. After the sharks were returned to normocapnic conditions after 24 h, alkalosis (high pH) persisted regardless of a substantial and rapid decline in bicarbonate concentration. Adjustment of pH to initial levels takes probably longer to allow cellular processes to adjust. The rapid recovery indicates the reversibility of this mechanism. Esbaugh *et al*.^26^ have hypothesised that species that are adapted to low-level hypercapnia may no longer rely on traditional short-term acid-base regulation and use morphological changes (gill permeability, diffusion distances) instead or in addition. In *H. edwardsii* this may be a contributing factor but has not been studied here.

It is important to note that acute physiological responses often differ distinctly from those to chronic exposure. In *H. edwardsii*, however, the response shown after acute exposure was maintained for a period of more than 60 days. In both treatments, plasma pH levels were very similar and there was no acidosis as could be expected if compensatory mechanisms cannot be maintained for prolonged periods. The increase in bicarbonate concentrations seen in the Henderson-Hasselbalch diagram makes it also apparent that, at least in part, increased bicarbonate levels are part of these mechanisms, ensuring that the outward *p*CO_2_ gradient (0.6 Torr) is maintained at a level found in normocapnia (0.8 Torr). However, long term compensation is likely to come at a cost: Lower metabolic rates, dissolution of hard structures such as shells and carapaces have been found^3,45,46^. Although metabolic depression^47^ is an adequate, reversible strategy to mitigate against short term hypercapnic exposure, the concomitant reduction in somatic growth and reproductive output might have negative effects during chronic exposure. This was previously shown in Port Jackson sharks^48^.

Structural- and compositional changes of denticles under chronic hypercapnic conditions were evident from structural scans and elemental composition of samples. Weakening and deformation of CaCO_3_ shell and skeleton elements has been observed in a host of different marine invertebrates^30^ and fishes^49^ and largely attributed to the negative effects of increased *p*CO_2_ on calcification or chemical dissolution. Shark denticles differ from invertebrate shells and skeletal structures as they are composed of calcium fluoro phosphate (fluorapatite) and calcium hydroxyl phosphate (hydroxyapatite)^37^. Although both materials are only weakly soluble^50^, the H^+^ concentration of 50 nM in our experiment seems to have been sufficient to dissolve a measurable portion of the apatites. This is evident from the lower concentration of Ca, P, and F in denticles exposed to those conditions (Table 4). The observed changes here are not the result of a physiological process, as the time it takes to form new denticles is in the order of 4 months^51^ and therefore exceeds the duration of the experiment. The observed effects are the result of chemical dissolution, but there is no information on such an effect on shark denticles under chronic hypercapnia. Green and Jutfelt^21^ found no visible change in *Scyliorhinus canicula* denticle morphology after short term (one month) hypercapnic exposure (pH 7.7). *S. canicula* and *H. edwardsii* are both common in areas with strong coastal upwelling and large fluctuations in seawater conditions, including temperature and pH, and are therefore likely adapted to acute changes in these. Our results however suggest that chronic exposure to severe hypercapnic (pH 7.3) conditions causes the dissolution of fluorapatite and in turn corrosion and weakening of the denticle surface. Shark denticles have been attributed a number of different functions, the protection against skin abrasions during hunting and mating and the improvement of hydrodynamics^52^. Our results suggest that chronic exposure to lower pH commensurate to future ocean acidification scenarios might impair the functionality of the denticles and in turn have negative effects for feeding and hydrodynamics. The latter might not be important in demersal sharks such as *H. edwardsii* that are buccal ventilators and do not need to swim in order to breathe. We speculate that, in large pelagic elasmobranchs which are facultative ram ventilators and must maintain forward motion to sufficiently ventilate their gill surface, impaired hydrodynamics by denticle corrosion may impact metabolic CO_2_ removal from the gills. However, further research is needed to examine this hypothesis. The intact denticle surface is responsible for as much as 12% increase in swimming speed^52^. Reduced hydrodynamics will not only make their swimming less effective, but will in addition make it more difficult to remove metabolic CO_2_. In a mackerel (teleost), higher swimming speed was shown to eliminate CO_2_ more efficiently from the blood^39^. In addition, corrosion of the teeth surface of all sharks will impair feeding.

## Conclusions

*H. edwardsii* are already well adapted to hypercapnic conditions due to the frequent occurrence of these after coastal upwelling and subsequent low-oxygen events. Despite these adaptations, we observed negative consequences during chronic hypercapnia: denticle corrosion. Denticle corrosion and the resultant increase in denticle turnover can potentially compromise hydrodynamics and skin protection. As denticles and shark teeth are structurally and materially identical, chemical dissolution of teeth at a similar rate can be expected. We speculate that a combination of these multiple effects might negatively affect the populations of this and other endemic, coastal elasmobranch species for which range shift is impossible as they reside at the southern tip of the African continent. We suggest that these multiple stressors make chondrichthyans particularly susceptible to ocean acidification and additional studies are urgently needed to elucidate the extent of this effect on already vulnerable species.
